# Plant-derived nanovesicles from *Ginkgo biloba* seeds mitigate LPS-induced endothelial dysfunction and promote vascular homeostasis

**DOI:** 10.3389/fbioe.2025.1715489

**Published:** 2026-02-13

**Authors:** Maneea Moubarak, Emese Szilágyi-Tolnai, Ani Barbulova, Immacolata Fiume, Ildikó Kovács-Forgács, Judit Homoki, Georgina Pesti-Asbóth, Endre Szilágyi, Ramila Mammadova, Matic Kisovec, Marjetka Podobnik, Dávid Papp, Gitta Schlosser, Judit Remenyik, Gabriella Pocsfalvi

**Affiliations:** 1 Extracellular Vesicles and Mass Spectrometry Laboratory, Institute of Biosciences and BioResources, National Research Council of Italy, Naples, Italy; 2 Complex Systems and Microbiome-Innovations Centre, Faculty of Agricultural and Food Sciences and Environmental Management, University of Debrecen, Debrecen, Hungary; 3 Department of Molecular Biology and Nanobiotechnology, National Institute of Chemistry, Ljubljana, Slovenia; 4 MTA-ELTE Lendület Ion Mobility Mass Spectrometry Research Group, Institute of Chemistry, ELTE Eötvös Loránd University, Budapest, Hungary

**Keywords:** endothelial inflammation, endothelial dysfunction, *Ginkgo biloba*, plant-derived nanovesicles, proteomics, vascular protection

## Abstract

**Background:**

Endothelial dysfunction is increasingly recognized as an early and central event in the onset of cardiovascular and neurodegenerative diseases. *Ginkgo biloba* extracts are known for their vascular-protective properties, including enhancement of endothelial function, antioxidant and anti-inflammatory activity, nitric oxide preservation, and modulation of platelet aggregation. Plant-derived nanovesicles (PDNVs) are emerging as versatile bioactive carriers with demonstrated anti-inflammatory, anticancer, antimicrobial, regenerative, and microbiota-modulating effects. However, their vascular-protective potential remains underexplored. This study aimed to assess the effects of PDNVs isolated from *Ginkgo biloba* seeds on endothelial responses under inflammatory stress.

**Methods:**

PDNVs were isolated from *Ginkgo biloba* seed homogenate using differential ultracentrifugation followed by density gradient ultracentrifugation with linear and non-linear iodixanol gradients. Nanoparticle tracking analysis (NTA) and cryo-transmission electron microscopy (cryo-TEM) characterized vesicle size, concentration, and morphology. Untargeted mass spectrometry profiled the protein content of distinct PDNV fractions. Functional assays were conducted on human umbilical vein endothelial cells (HUVECs) exposed to lipopolysaccharide (LPS)-induced inflammatory stress.

**Results:**

Ginkgo PDNV isolates comprise a heterogeneous population of nanometer sized particles, including vesicles with single and double layers. Proteomics revealed seed storage proteins (legumin and ginnacin) and membrane-associated ATPases, HSP90, catalase, phosphoenolpyruvate carboxylase (PEPC), and eEF1A. PDNVs were non-toxic up to 50 μg/mL; at 100 μg/mL, they enhanced mitochondrial activity but triggered early apoptosis and necrosis. PDNVs did not increase ROS production, even in the presence of H_2_O_2_. At 1 μg/mL, they significantly suppressed LPS-induced expression of IL-1β, TNF-α, IL-6, and IL-8 (mRNA and protein; p ≤ 0.05 to p ≤ 0.001). PDNVs preserved endothelial integrity by downregulating VCAM-1 and upregulating occludin, maintained eNOS expression (p ≤ 0.01), and attenuated COX-1, COX-2, and prostacyclin synthase (PGIS) induction. Thrombotic markers (TXB_2_, vWF, and PAI-1) remained unaffected.

**Conclusion:**

Ginkgo seed-derived PDNVs exhibit vascular-protective and anti-inflammatory properties, supporting their potential as safe, multifunctional agents for endothelial modulation. Further studies are warranted to explore their therapeutic applications in vascular biology.

## 1 Introduction

The endothelium is a metabolically active monolayer of cells lining the inner surface of blood vessels, acting as a dynamic barrier between circulating blood and surrounding tissues. It plays a central role in maintaining vascular homeostasis and adapting to environmental stimuli by producing a wide array of signaling molecules (Zhou et al., 2023). Key endothelial functions include regulation of vascular permeability, vasomotor tone, inflammation, cell proliferation, low-density lipoprotein (LDL) oxidation, and coagulation balance (Michiels, 2003). Endothelial dysfunction (ED) arises when vascular homeostasis is disrupted by risk factors such as hypertension, oxidized LDL, and diabetes (Yang et al., 2024). ED is characterized by impaired nitric oxide (NO) bioavailability, elevated reactive oxygen species (ROS), and increased vascular inflammation. This pro-inflammatory phenotype involves upregulated cytokines (IL-1, IL-6, IL-18, TNF-α, and CRP) (Dri et al., 2023), which enhance adhesion molecule expression (ICAM-1 and VCAM-1), compromise endothelial barrier integrity, and promote monocyte transmigration (García-Ponce et al., 2016). Chronic vascular inflammation is a key driver of atherosclerosis and links cardiovascular diseases (CVD) with neurodegenerative disorders (Hansson, 2005). The global rise in non-communicable diseases is driven by aging populations, sedentary lifestyles, obesity, and metabolic conditions such as diabetes and hyperlipidemia. These trends underscore the need for effective, low-cost adjunct strategies to complement conventional therapies and mitigate endothelial oxidative and inflammatory stress.

Plant-derived nanovesicles (PDNVs) have emerged as valuable biomaterials in the field of extracellular vesicle (EV) research. However, their definition and nomenclature remain inconsistent, underscoring the need for standardization to ensure clarity and reproducibility (Pinedo et al., 2021; Tinnirello et al., 2023). The Plant EVs Task Force of the International Society for Extracellular Vesicles (ISEV) recommends using “extracellular vesicle” as a generic term exclusively for the naturally released, lipid bilayer-enclosed vesicles secreted by plant cells. In contrast, vesicles obtained through disruptive isolation methods, often containing intracellular components, should be referred to as “plant-derived nanovesicles” (PDNVs) to acknowledge their mixed origin. Building on this distinction, it is also important to recognize that canonical EV markers established in model systems such as Arabidopsis, most notably the tetraspanin TET8, as well as recurring proteins such as aquaporins, HSP70, and 14-3-3 proteins, are not consistently detected across plant species or tissues. Recent cross-species proteomic analyses have further proposed *bona fide* plant EV markers, including fasciclin-like arabinogalactan proteins (FLAs), germin-like proteins (GLPs), and patellins (PATs), highlighting partial proteome conservation between monocots and eudicots (Rodríguez de Lope et al., 2025). Consensus criteria for plant EV markers remain under development, and variability in detection across species and tissues has been highlighted in recent reviews (Sall and Flaviu, 2023). PDNVs isolated from homogenized tissues, including seeds, are heterogeneous preparations that typically lack these canonical markers, reflecting their mixed origin and compositional complexity. Rather than a limitation, this complexity represents an opportunity to work with a multifaceted biomaterial that encompasses EVs together with other vesicular and non-vesicular structures, offering diverse cargo profiles and functional potential. PDNVs are efficiently internalized by recipient cells, as supported by recent studies, enabling delivery of diverse vesicular and non-vesicular cargo (Lin et al., 2025).

PDNV research has predominantly focused on leaf, root, and fruit tissues, which have been widely studied for their bioactive cargo and therapeutic potential (Pocsfalvi et al., 2018; Di Gioia et al., 2020; Stanly et al., 2020; Chen et al., 2023; Mammadova et al., 2023; 2025; Sall and Flaviu, 2023; Chai et al., 2025; Jung et al., 2025; Zheng et al., 2025). Seeds have only recently become a focus of PDNV research, especially in leguminous species. Notably, PDNVs from *Phaseolus vulgaris* (common bean) have been shown to modulate gut microbiota and mitigate colitis through immune regulatory mechanisms (Pang et al., 2024). Additional studies have purified PDNVs from peas and soybeans (Xiao et al., 2018; Alkhaldi et al., 2024). Soybean PDNVs facilitate improved topical delivery of curcumin, improving its skin bioavailability and therapeutic effectiveness. Despite these advances, PDNVs from seeds of arboreal species, including conifers, cycads, and *Ginkgo biloba* (GB), remain largely uncharacterized. This gap highlights the potential of tree seed-derived PDNVs as a promising yet underexplored frontier in nanovesicle research, with implications for nutrition, immunomodulation, and sustainable biotherapeutics.

Seeds vary widely in chemical composition, including moisture, protein, lipids, ash, sugars, tannins, and phytates and offer a rich biochemical landscape for vesicle cargo formation. As PDNV research expands, seeds with known medicinal or nutritional properties are increasingly recognized for their unique molecular profiles. Compared with mammalian vesicles, PDNVs have been reported to exhibit lower immunogenicity, reduced risk of zoonotic pathogen transmission, and minimal environmental contamination during isolation. These literature-based safety advantages provide a rationale for investigating novel sources such as GB seeds (Logozzi et al., 2022; Duraisamy et al., 2024; Hillman, 2024).

GB seeds are renowned for their diverse secondary metabolites, including ginkgolides, bilobalide, and ginkgotoxin (Biernacka et al., 2023; Árva et al., 2024). Ginkgolides are potent platelet-activating factor (PAF) antagonists with anti-inflammatory and neuroprotective effects, while bilobalide modulates neurotransmission and protects against ischemic damage. In contrast, ginkgotoxin (4′-O-methylpyridoxine) can interfere with vitamin B6 metabolism, posing neurotoxic risks when consumed in excess. Accurate profiling of these compounds is essential for both therapeutic and safety considerations (Árva et al., 2024). Despite this well-characterized metabolite composition, PDNVs derived from GB seeds remain uncharacterized, leaving a critical gap in understanding their structural features, cargo profiles, and biological functions. Addressing this gap is essential to determine whether GB seed PDNVs share the bioactive properties of their parent tissue and to explore their potential applications in vascular health and disease.

In this study, we developed and optimized protocols to isolate PDNVs from GB seed homogenate using differential ultracentrifugation (DUC), targeting both low- and high-velocity fractions. These were further purified via iodixanol density gradient ultracentrifugation (DGUC), employing both linear and non-linear gradients to resolve vesicle populations by density. Cryo-transmission electron microscopy (cryo-TEM) was used to assess vesicle morphology and membrane structure, while nanoparticle tracking analysis (NTA) quantified vesicle concentration and yield (particles per gram of ginkgo seed). We also performed untargeted, mass spectrometry-based proteomics on PDNVs obtained from DUC- and DGUC-separated fractions. We then designed a series of *in vitro* assays using human umbilical vein endothelial cells (HUVECs), one of the most widely used cell models in EV functional assays, especially when studying vascular biology, inflammation, and angiogenesis, to evaluate the potential of ginkgo PDNVs to modulate endothelial responses under inflammatory stress. These assays aim to assess cytotoxicity, mitochondrial activity, oxidative stress, cytokine expression, and markers of endothelial integrity and vascular homeostasis. This approach helped us elucidate the functional relevance of GB PDNVs in vascular protection and provided insights into their potential as safe, multifunctional agents for inflammation-related vascular disorders.

## 2 Materials and methods

### 2.1 Seed material and PDNV isolation

GB matured fruits were collected from two trees in the Piscinola public garden, Naples, in October 2022. The fleshy sclerotestae were removed, and the seeds were washed several times under tap water. The outer stony layer (seed coat, mesoderm) was removed using a nutcracker, and the brown membranous inner seed coat (endopleura) covering the endosperm was also removed with a scalpel. Only the raw kernels (pericarp) were used for these experiments. Extractions of ginkgo seeds were performed using 20–50 nuts corresponding to 30–60 g of peeled seeds. Seeds were washed with tap water, then with MilliQ water. Homogenization was performed in 300–600 mL 0.05 M citrate buffer, pH 6, using a kitchen blender homogenizer. The DUC method, as previously described (Bokka et al., 2020), was used to isolate two fractions from ginkgo homogenates with some differences. In brief, low-velocity centrifugation steps were performed at 400 × *g*, 800 × *g*, and 2,000 × *g* using a swinging-bucket rotor (Eppendorf AG centrifuge 5804 R, Hamburg, Germany) and 15,000 × *g* using a fixed-angle bucket rotor (Eppendorf AG centrifuge 5804 R, Hamburg, Germany) for 30 min at 24 °C. Supernatant obtained in the 15,000 × *g* step (S15k) was analyzed by light microscopy (Leica DMi8 reversed microscope) for the presence of observable structures. Following the centrifugation at 15,000 × g, micrometer-sized structures were observed in the resulting supernatant (S15k) via light microscopy. To remove residual particulates, the S15k fraction was subsequently filtered through glass filter paper and re-examined by light microscopy prior to ultracentrifugation. The pellets obtained from the 15,000 × g step (P15k) were collected and pooled. Ultracentrifugation of the S15k fraction was performed at 100,000 × g for 120 min at 4 °C using a Type SW 28Ti rotor in a Beckman Optima XL-A analytical ultracentrifuge (Beckman Coulter, California, United States). The resulting pellet (P100k) was resuspended in 0.05 M citrate buffer (pH 6) and washed by ultracentrifugation under the same conditions. The supernatant obtained from the 100,000 × g centrifugation step (S100k) was examined by light microscopy using a Leica DMi8 inverted microscope. To concentrate protein components, aliquots of the supernatants from the 2,000 × g (S2k; 1 mL) and 100,000 × g (S100k; 10 mL) centrifugation steps were precipitated by the addition of ice-cold acetone, followed by overnight incubation at −20 °C. Pellets were collected and further analyzed.

### 2.2 Gradient density ultracentrifugation

**FIGURE 1:**
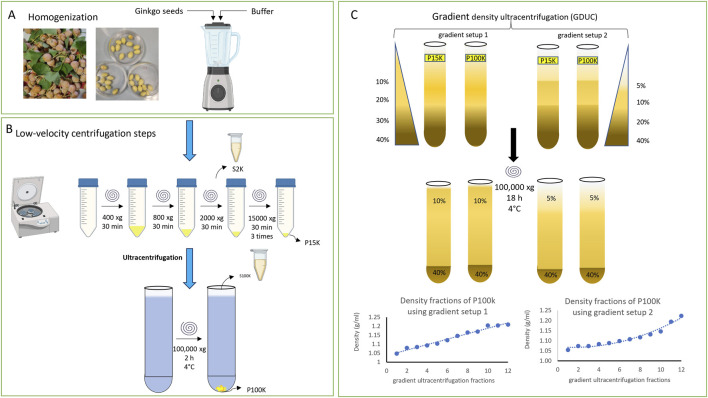
Schematic workflow illustrates the isolation of *Ginkgo biloba* seed-derived vesicles, including **(A)** homogenization, **(B)** low-speed centrifugation, and ultracentrifugation steps. Pellets obtained at 15,000 × g and 100,000 × g were designated as P15k and P100k, respectively. The P15k and P100k fractions were subsequently purified and fractionated using iodixanol gradient density ultracentrifugation (GDUC) - panel C. Discontinuous iodixanol gradients composed of either 10%, 20% 30%, and 40% (setup 1) or 5%, 10%, 30%, and 40% (setup 2) layers of iodixanol were prepared for density-based separation **(C)**. The bottom graphs show the densities measured in each fraction of P100k collected after the DGUC step in the two different discontinuous gradient setups.

The P15k and P100k samples were purified by density gradient ultracentrifugation (DGUC) using OptiPrep™ (iodixanol; Abbott Diagnostics Technology AS, Oslo, Norway) as the density gradient medium. Gradient cushions of 10%, 20%, 30%, and 40% (v/v) and 5%, 10%, 20%, and 40% (v/v) iodixanol were sequentially underlaid, and the respective samples were carefully layered on top in an open-top, thin wall polypropylene 38.5 mL capacity UC tube (Beckman Coulter, Inc., Brea, CA, United States) (Figure 1B). Ultracentrifugation was performed at 100,000 × g for 18 h at 4 °C using a Type SW 41Ti rotor (Beckman Coulter, California, United States).

Following ultracentrifugation, 12 sequential 1 mL fractions (F1–F12) were collected from the top to the bottom of the gradient, with a final 500 μL fraction (F12) obtained for both P15k and P100k sample sets. To eliminate residual iodixanol, each DGUC fraction was washed twice with extraction buffer by ultracentrifugation at 100,000 × g for 2 h at 4 °C. The resulting pellets were resuspended in a minimal volume of extraction buffer. Samples were either processed immediately or stored at −80 °C for subsequent analysis.

### 2.3 Density determination, protein and lipid quantification, and SDS-PAGE analysis

The density of each fraction was measured based on the iodixanol concentration obtained by a NanoDrop 2000 spectrophotometer (Thermo Fisher Scientific Inc., Waltham, MA, United States) at 244 nm wavelength using a six-point calibration curve. Measurements of the standards to create the calibration curve and the samples were performed according to Bokka et al. (2020).

The protein concentration was determined by the Qubit assay. A three-point calibration curve was performed according to the manufacturer’s instructions using the Qubit™ Standard reagents (Thermo Fisher Scientific, Singapore, United States). For the estimation, 1 µL of the sample was combined with 199 µL Qubit™ Working Solution, and protein concentration was measured on Qubit™ 4 fluorometer (Thermo Fisher Scientific, Singapore, United States).

The colorimetric reaction of sulfuric acid and phospho-vanillin with lipids (SPV assay) was performed in a 96-well plate format to quantify the unsaturated fatty acids. The method was modified from Visnovitz et al. (2019). Lipid standard solutions at 2 μg/μL were prepared from cholesterol (Sigma-Aldrich, Darmstadt, Germany)/menhaden fish oil (Sigma-Aldrich, Darmstadt, Germany) in chloroform (Romil, Deltek, Pozzuoli, Italy), and different volumes (0 μL, 1 μL, 2 μL, 4 μL, 7 μL, and 14 μL) of standard solutions were pipetted into glass reaction vials. PDNV samples containing 10 μg of protein were dried in a 1.5 mL Eppendorf tube using a vacuum concentrator (Savant SpeedVac SC100, Farmingdale, NY, United States). A small volume of chloroform was added to the dried samples, vortexed, and transferred to glass vials. Chloroform was added to standard solutions and samples in each vial up to 14 μL final volume. The chloroform was evaporated by incubating the vials at 60 °C in a heater block (Reacti-Therm™ Heating and Stirring Module TS-18822, Thermo Fisher Scientific). A 200 μL aliquot of 96% sulfuric acid (Sigma-Aldrich, Darmstadt, Germany) was added to all standards and samples in glass vials. The vials were incubated with open lids at 90 °C for 20 min in a heater block and cooled to room temperature by placing them at 4 °C for at least 5 min. A 120 μL aliquot of phospho-vanillin reagent [the solution of vanillin (Sigma-Aldrich, St. Louis, MO, United States) in 17% phosphoric acid (Deltek, Pozzuoli, Italy), 1 mg/mL] was added to each vial and vortexed. A 280 μL aliquot of each sample was transferred to a 96-well plate (Techno Plastic Products, Switzerland) and incubated for 1 h at 37 °C to develop the colorimetric reaction. Absorbance was measured at 520 nm using a microplate reader (AMR-100, Hangzhou Allsheng Instruments Co., Ltd., China), and lipid content was subsequently quantified.

Sodium dodecyl sulfate polyacrylamide gel electrophoresis (SDS-PAGE) was performed to separate proteins and obtain protein profiles. Novex Bolt 4%–12% Bis-Tris Plus gel (Invitrogen, Carlsbad, CA, United States) and Bolt MOPS SDS running buffer (Invitrogen, Carlsbad, CA, United States) were used according to the manufacturer’s instructions. Proteins were separated using 165 V for 45 min. Gel was washed with water, fixed in 40% (v/v) methanol and 10% (v/v) acetic acid for 1 h, and stained with colloidal Coomassie Brilliant blue G-250 (AppliChem GmbH Darmstadt, Germany). Destaining was performed using Milli Q water.

### 2.4 Nanoparticle tracking analysis (NTA)

Particle size distribution was assessed using nanoparticle tracking analysis (NTA) with a NanoSight NS300 system (Malvern Technologies, Malvern, United Kingdom), equipped with a 488 nm laser and a high-sensitivity scientific CMOS camera. Prior to analysis, samples were diluted to a volume of 1 mL with particle-free phosphate-buffered saline (PBS) filtered using a 0.22 µm filter to achieve optimal particle concentration. Measurements were conducted under constant flow conditions (flow rate = 50 arbitrary unit (a.u.)) at 25 °C. Data acquisition and analysis were performed using NanoSight NTA software version 3.4. In selected experiments, PDNV samples were sonicated in an ultrasonic bath for 2 min to disperse potential aggregates.

### 2.5 Cryogenic transmission electron microscopy (cryo-TEM)

C-flat™ 2/2, 200 mesh holey carbon grids (Protochips, Morrisville, NC, United States) were glow-discharged using a GloQube® Plus system (Quorum Technologies, Laughton, United Kingdom) under the following conditions: 20 mA current, 60 s duration, positive polarity, and ambient air atmosphere. A 2 µL aliquot of the sample was applied to each grid, blotted immediately, and vitrified in liquid ethane using a Vitrobot Mark IV (Thermo Fisher Scientific, Waltham, MA, United States). Vitrobot settings were configured to 100% relative humidity at 4 °C, with a blot force of 4 and a blot time of 6 s. Grids were imaged under cryogenic conditions using a 200 kV Glacios transmission electron microscope equipped with a Falcon 3EC direct electron detector (Thermo Fisher Scientific, Waltham, MA, United States).

### 2.6 Lysis and in-solution trypsin digestion of the samples

Samples were dried and suspended in 10 µL of 0.2% (w/v) RapiGest™ SF Surfactant (Waters Corporation, Milford, MA, United States) solution prepared in 50 mM ammonium bicarbonate (AMBIC). Vesicle lysis was performed using five freeze-thaw cycles, with freezing in liquid nitrogen and thawing under sonication for 1-2 minutes per step. Proteins were reduced by 5 mM dithiothreitol (DTT, Sigma-Aldrich, Saint Louis, MO, United States) in 50 mM AMBIC and alkylated using 15 mM iodoacetamide (Sigma-Aldrich, Saint Louis, MO, United States) in 50 mM AMBIC. Proteins were proteolytically digested using mass spectrometry-grade trypsin (Pierce, Thermo Fisher Scientific, Rockford, IL, United States) at a 1:100 protein:trypsin ratio at 37 °C overnight. RapiGest was cleaved by acidifying the samples with trifluoroacetic acid to a final concentration of 0.5% (v/v) and then centrifuging at maximum velocity for 10 min. The supernatant containing the tryptic peptides was vacuum dried.

### 2.7 LC-ESI-MS/MS analysis

Shotgun proteomic analysis by liquid chromatography–electrospray ionization tandem mass spectrometry (LC–ESI-MS/MS) was performed as previously described (Mammadova et al., 2023). Prior to analysis, samples were purified using Pierce C18 Spin Columns (Thermo Fisher Scientific, Waltham, MA, United States) according to the manufacturer’s instructions. Eluted peptides were dried using a SpeedVac concentrator and stored at −20 °C. Before MS analysis, dried samples were reconstituted in a solution of 2% acetonitrile and 98% water (v/v), containing 0.1% (v/v) formic acid. Mass spectrometric measurements were conducted using a high-resolution hybrid quadrupole–time-of-flight instrument (Waters Select Series Cyclic IMS, Waters Corp., Wilmslow, United Kingdom) equipped with a low-flow electrospray ionization source. Peptide separation was achieved using a Waters Acquity I-Class ultra-performance liquid chromatography (UPLC) system directly coupled to the mass spectrometer. Chromatographic separation was performed on a Waters Acquity CSH Peptide C18 column (1 mm × 150 mm, 1.7 µm particle size). Gradient elution was carried out under the following conditions: eluent A—0.1% formic acid in water; eluent B—0.1% formic acid in acetonitrile; flow rate—20 μL/min; column temperature—45 °C; gradient profile—1 min: 5% B, 45 min: 35% B, 46 min: 85% B.

MS data acquisition was performed in V-mode over an *m/z* range of 50–2,000, with a scan time of 0.5 s. Leucine enkephalin was used as a single lock mass for calibration. Fragmentation was conducted in the trap region using low energy (6 V) and ramped high energy (19–45 V).

Raw data were processed using ProteinLynx Global Server version 3.0.3 (Waters Corp., Wilmslow, United Kingdom). Background noise was reduced using the Compression Tool version 1.10 (Waters Corp., Wilmslow, United Kingdom), with a threshold set to 10 ion counts. Data processing parameters included: low energy threshold: 200 counts; increased energy threshold: 20 counts; minimum fragment ion matches per peptide: 3; minimum fragment ion matches per protein: 7; and minimum peptide matches per protein: 2.

### 2.8 *In vitro* bioassays

#### 2.8.1 Cell culture conditions

We used immortalized human umbilical cord vein endothelial cells (HUVEC/TERT2) obtained from ATCC (Manassas, VA, United States) for the experiments. Cells were cultured according to the manufacturer’s instructions. HUVECs were maintained in M199 medium supplemented with 10% heat-inactivated FBS, 1% penicillin/streptomycin, 1% amphotericin B, 2 mM glutamine, and Endothelial Cell Growth Medium-2 (EGM-2) at 37 °C in a humidified incubator under 5% CO_2_. Media was changed every 48 h until cells reached 80%–90% confluency. At confluency, cells were either sub-cultured or used for experiments. All experiments were performed with cells at passage 16. The same medium composition was used as a control. Prior to seeding, a 0.1% gelatin solution was applied to support cell adhesion. To establish the inflammatory model, LPS (eBioscience, San Diego, CA, United States) was added to M199 medium at a final concentration of 200 ng/mL.

#### 2.8.2 Determination of cell viability

Cell viability was assessed using the MTT (3-[4,5-dimethylthiazol-2-yl]-2,5 diphenyltetrazolium bromide) assay, which measures the formation of formazan crystals from tetrazolium salts by mitochondrial dehydrogenases. Cells were seeded in 96-well plates at a density of 20,000 cells/well and treated with different concentrations of PDNVs (100 μg/mL, 50 μg/mL, 10 μg/mL, 5 μg/mL, 1 μg/mL, and 0.1 μg/mL) for 24 h and 48 h. Following treatment, cells were washed with PBS and incubated with 0.5 mg/mL MTT solution for 3 h. Formazan crystals were formed in proportion to the number of viable cells. They were subsequently dissolved in 100 µL/well of solubilizing solution 81% (v/v) isopropyl alcohol (Serva, Heidelberg, Germany), 10% (v/v) Triton X-100 (Serva, Heidelberg, Germany), and 9% (v/v) 1 M HCl (Serva, Heidelberg, Germany). Absorbance was at 465 nm using a CLARIOstar microplate reader (BMG Labtech, Ortenberg, Germany). Results were expressed relative to the control group.

#### 2.8.3 Determination of apoptosis

The mitochondrial membrane potential of HUVECs was assessed using the fluorescent dye 1,1′,3,3,3′,3′-hexamethylindodicarbocyanine iodide (DilC1(5)). A decrease in DilC1(5) fluorescence intensity indicated mitochondrial depolarization, which is a hallmark of early apoptotic processes in HUVECs.

Cells were seeded in black 96-well plates at a density of 20,000 cells per well and treated with PDNVs at various concentrations (100 μg/mL, 50 μg/mL, 10 μg/mL, 5 μg/mL, 1 μg/mL, and 0.1 μg/mL). After treatment, the medium was removed, and cells were incubated for 30 min with 50 µL per well of DilC1(5) working solution (50 nM in Dulbecco’s Modified Eagle’s Medium). Following incubation, cells were washed twice with PBS, and fluorescence was measured using a CLARIOstar microplate reader (BMG Labtech, Ortenberg, Germany) at excitation and emission wavelengths of 630 nm and 670 nm, respectively.

#### 2.8.4 Determination of necrosis

Necrotic processes were assessed using SYTOX Green staining. This membrane-impermeable dye selectively penetrates cells with compromised plasma membranes, binding to nucleic acids and emitting fluorescence, whereas intact, viable cells exclude the dye and exhibit minimal fluorescence.

Cells were cultured in 96-well plates and treated as indicated with PDNVs at various concentrations (100 μg/mL, 50 μg/mL, 10 μg/mL, 5 μg/mL, 1 μg/mL, and 0.1 μg/mL). After treatment, the medium was removed, and cells were incubated for 30 min with 50 μL/well SYTOX Green dye (1 μM dissolved in Dulbecco’s modified Eagle’s medium), followed by washing with PBS. The fluorescence of SYTOX Green was measured at 490 nm excitation and 520 nm emission wavelengths by using a CLARIOstar microplate reader (BMG Labtech, Ortenberg, Germany). The results were expressed relative to 100% of the control group.

#### 2.8.5 Measurements of pro-inflammatory cytokine protein expression

HUVECs were seeded into a six-well plate (600,000 cells/well) and incubated with 1 μg/mL PDNVs (µg/mL) alone or in combination with LPS (200 ng/mL). Supernatants were collected, centrifuged for 10 min at 10,000 rpm, and the concentrations of IL-6 and IL-8 were determined using a Human ELISA kit (Thermo Fisher Scientific, MA, United States) according to the manufacturer’s instructions.

#### 2.8.6 Reactive oxygen species (ROS)

HUVECs were seeded in a 96-well plate. Intracellular ROS were labeled by incubating the cells with 100 μMol of 2′,7′-dichlorodihydrofluorescein diacetate (DCFDA) for 1 h at 37 °C. Following incubation, cells were washed twice with PBS and subsequently treated with 100 μM of H_2_O_2_. M199 medium was used as the control. PDNVs were applied at different concentrations (100 μg/mL, 50 μg/mL, 10 μg/mL, 5 μg/mL, 1 μg/mL, and 0.1 μg/mL). ROS levels were quantified by measuring DCFDA fluorescence intensity, which is proportional to ROS production, using a CLARIOstar microplate reader (BMG Labtech, Ortenberg, Germany). Results were expressed relative to the control group.

#### 2.8.7 Measurement of gene expression by qRT-PCR

For mRNA analysis, HUVECs were transferred into six-well plates and pre-treated with PDNVs (1 μg/mL) for 16 h. We included a negative control (PDNVs alone), lipopolysaccharides (LPS) alone at 200 ng/mL, and a combined treatment with LPS and PDNVs. After the PDNV pretreatment, LPS was added to induce inflammation in human umbilical vein endothelial cells (HUVECs). Total mRNA was extracted with RNeasy Mini Kit (Qiagen, Milano, Italy) and quantified by spectrophotometric analysis at 260 nm using a NanoDrop ND-100 spectrophotometer (Euroclone, Italy).

Reverse transcription was performed with 1 ng of total RNA using a TaqMan™ cDNA Synthesis Kit (Thermo Fisher Scientific, United States). Quantitative real-time PCR with Gene Expression Assays (Thermo Fisher Scientific, United States) was performed on a LightCycler® 480 Real-Time PCR System (Roche Diagnostics, Risch-Rotkreuz, Switzerland). Each reaction included negative controls (no template for RNA isolation and reverse transcription) and was run in triplicate. GAPDH was used as the reference gene for normalization. Mean Ct values were calculated, and relative gene expression was determined using the comparative ΔCt method according to the Livak protocol.

#### 2.8.8 Statistical analysis

Statistical analysis was performed with the GraphPad statistical software package (GraphPad Software, La Jolla, CA). Statistical probes were based on two-way ANOVA with the Bonferroni post-hoc test. p < 0.05 was considered statistically significant. Data are presented as mean ± standard deviation (SD). The data were analyzed and presented using GraphPad Prism 8.3.1 (GraphPad Software, La Jolla, CA, United States). The data are presented as mean ± SD. Differences were considered statistically significant when p < 0.05. Asterisks report statistical significance ***p < 0.001.

## 3 Results

### 3.1 Isolation of PDNVs from *Ginkgo biloba* seeds

PDNVs were isolated from GB seeds following the workflow illustrated in Figure 1, which includes three main steps: homogenization (Figure 1A), differential ultracentrifugation (DUC; Figure 1B), and density gradient ultracentrifugation (DGUC Figure 1C). Homogenization was performed using a 50 mM citrate buffer (pH 6.0). The resulting homogenate was subjected to stepwise DUC, involving centrifugation at increasing speeds to separate components based on size and density. After the 2,000 × g step, a small amount of supernatant (S2k) was taken for analysis, and the rest was progressed. Centrifugation at 15,000 × g yielded a pellet (P15k), while the supernatant (S15k) was examined by light microscopy to assess the presence of residual micrometer-sized particles. The continued presence of large particles in the S15k fraction indicated incomplete removal of co-purifying material. Therefore, the 15,000 × g centrifugation step was repeated until no further large particles were observed. Subsequent ultracentrifugation at 100,000 × g produced a second pellet, which was resuspended in a small volume of isolation buffer and purified using an additional ultracentrifugation step. This washed P100k pellet represented the second PDNV sample (Figure 1B).

**FIGURE 2:**
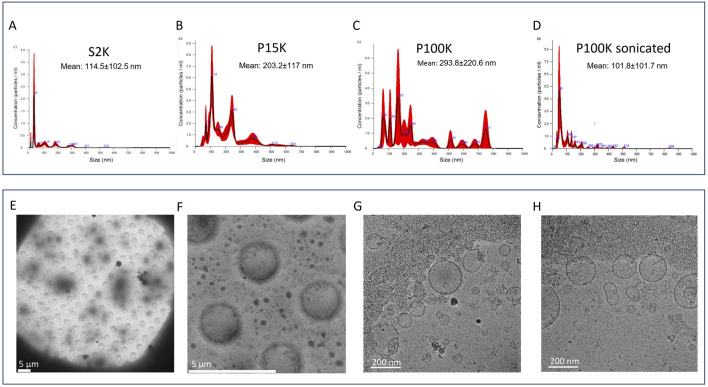
Size distribution and morphological characteristics of *Ginkgo biloba* seed-derived nanovesicles. The upper panel **(A–D)** shows the results of the nanoparticle tracking analysis (NTA) performed on samples obtained at selected steps of the isolation process (Figure 1): **(A)** supernatant after 2,000 × *g* centrifugation (S2k), **(B)** pellet after the 15,000 × *g* centrifugation (P15k), **(C)** pellet after the 100,000 × *g* ultracentrifugation (P100k), and **(D)** P100k sample after sonication. Mean particle size ± standard deviation (SD) indicated in nm. The lower panel **(E–H)** shows the cryo-TEM images of a vitrified P100k sample at various magnifications: **(E)** low-magnification image of one single grid square at ×700, **(F)** medium magnification at ×6,700 magnification, and **(G, H)** representative images of the preparation at ×57,000 magnification. Scale bars: 5 μm for **(E, F)** and 200 nm for **(G, H)**.

NTA was performed to monitor the PDNV isolation process at various stages (Figure 1A). The S2k supernatant (prior to P15k isolation) and the S100k supernatant (following the isolation of both P15k and P100k) were analyzed alongside the P15k and P100k PDNV samples. The S2K sample exhibited particles with a mean diameter of 114 nm (Figure 2A), accompanied by a high standard deviation (102.5 nm), indicative of pronounced polydispersity. Given that reliable tracking of biological particles by NTA is typically limited to sizes above 50–70 nm, the presence of a distinct peak at approximately 35 nm is noteworthy and may reflect the formation of protein aggregates. Ginkgo seeds are known to be rich in proteins, particularly ginkgo seed proteins (GSPs), which have molecular weights approximately 17 kDa. These proteins are prone to aggregation during extraction, especially under stress conditions such as shear forces, thermal exposure, pH fluctuations, or freeze–thaw cycles. Supporting this interpretation, the S2K sample also showed increased protein and particle concentrations (Table 1). NTA of the P15k and P100k samples revealed a broad and heterogeneous size distribution (Figures 2B,C). Given that PDNVs are prone to aggregation during isolation, particularly under ultracentrifugation conditions, the P100k sample was subjected to a short sonication in an ultrasonic bath. Particle concentration and size distribution were compared before and after sonication (Figures 2C,D). Following sonication, the mean particle size decreased by nearly two-thirds, from 293 nm to 101 nm, while particle concentration increased significantly from 4.83 × 10^10^ to 2.67 × 10^11^ particles/mL (Table 1). These results indicate that the ginkgo PDNV isolates contained a substantial number of particles within the NTA detection range, typically 30–1,000 nm, depending on particle type, refractive index, and instrument settings. Moreover, sonication of P100k effectively disrupted larger aggregates, enhancing particle dispersion, resulting in a more uniform and stable suspension of individual particles.

**TABLE 1:** The yield of *Ginkgo biloba* seed-derived plant-derived nanovesicles (PDNVs) was assessed based on protein concentration (measured by Qubit assay), particle concentration (determined via nanoparticle tracking analysis, NTA), and unsaturated fatty acid quantity (vanillin assay).

Sample	Protein quantity (mg/gram seed)	Particle concentration (particles/mL)	Unsaturated fatty acid quantity (mg/μg of protein)
S2k	9.3 ± 1.7	4.09E+11 ± 1.61E+11	1.92 ± 0.49
P15k	2.4 ± 0.3	2.39E+10 ± 8.30E+08	10.93 ± 1.93
P100k	0.28 ± 0.003	4.83E+10 ± 9.18E+09*2.67E+11 ± 1.18E+11	21.63 ± 2.96
S100K	6.0 ± 0.9	2.85E+11 ± 2.32E+10	1.40 ± 0.23

Protein quantities were normalized per gram of starting ginkgo seed material, particle concentrations were reported as measured in the respective samples, and fatty acid quantity was normalized to 1 μg of protein (measured by the Qubit assay). Analyses were performed on low-speed (P15k) and ultracentrifugation (P100k) pellets resolubilized in buffer, as well as on the supernatants collected after centrifugation at 2,000 × g (S2k) and 100,000 × g (S100k). Proteins in the S2k and S100k fractions were precipitated using acetone prior to quantification. *Particle concentrations in the P100K sample were obtained by NTA, following brief sonication to ensure dispersion. All measurements of protein and fatty acid content were conducted in triplicate using independent biological replicates.

**FIGURE 3:**
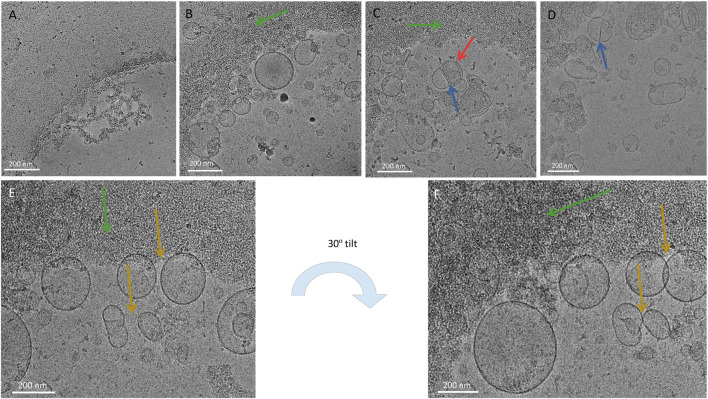
Cryo-TEM micrographs of P100k PDNV samples. **(A)** P100k sample before the repurification step. **(B–F)** Representative images show vesicles of varying sizes and morphologies embedded in vitreous ice. Multilamellar structures (red arrows), vesicle–vesicle contacts (blue arrows), and amorphous material aggregating near carbon foil edges (green arrows) are visible. **(E–F)** Tilted imaging (0° and 30°) highlights spatial relationships among vesicles, including invaginated profiles and apparent overlaps due to projection effects. Scale bars: 200 nm.

Cryo-TEM analysis confirmed the presence of vesicular structures exhibiting highly heterogeneous morphology and size (Figures 2E–H, 3B–F). In addition to vesicles, non-vesicular material was also detected. Notably, cryo-TEM imaging of the unwashed P100k samples failed to reveal distinct particles, likely because the abundance of co-purifying contaminants obscured vesicle visualization (Figure 3A). Compared to the unwashed sample, the purified P100k preparation (Figures 2E–H, 3B–F) was enriched in vesicles and displayed a broad range of structural features. Most vesicles were larger than 50 nm in diameter, with some reaching several hundred nanometers. Structural diversity included multilamellar vesicles (red arrow, Figure 3C) and vesicles forming close membrane contacts (blue arrows, Figures 3C,D). Additionally, a substantial amount of amorphous material, likely protein aggregates, was observed. These aggregates tended to cluster and preferentially localize along the edges of the carbon foil holes, suggesting potential interactions with the support film (Figures 2G,H; green arrows in Figures 3C,E,F).

To further investigate vesicle morphology, a pair of micrographs was collected at 0° and 30° tilt from the same position on the electron microscopy (EM) grid (Figures 3E,F). This dual-angle approach provided enhanced spatial context for an unusual vesicle located centrally (yellow arrow), which appeared invaginated on one side but otherwise retained the characteristics of a typical vesicle, with no signs of fusion or degradation. The tilted image also revealed two vesicles that seemed to overlap (right panel); however, the 0° image confirmed they were not in contact, and the apparent overlap was an artifact of the grid tilt. This observation supports the notion that vesicles are generally frozen in a single plane, rather than stacked above or below one another. Consequently, when a smaller vesicle is seen entirely enclosed within a larger one, it likely represents a true multilamellar vesicle (MLV) rather than a coincidental projection. The frequent occurrence of fully enclosed smaller vesicles further reinforces the MLV interpretation.

The particle concentration, protein yield, and unsaturated fatty acid content of the two PDNV samples (P15k and P100k), as well as the soluble fractions S2k (which contains both types of PDNVs) and S100k (depleted of both types), are reported in Table 1. Notably, although the protein content was higher in the P15k fraction (2.4 mg/g ± 0.3) than in the P100k fraction (0.28 mg/g ± 0.003), the latter exhibited a greater amount of unsaturated fatty acids. The P100k fraction, obtained via ultracentrifugation, typically isolates smaller, membrane-rich vesicles that contain less protein per particle than larger microvesicles. The increased unsaturated fatty acid content in P100k suggests a higher proportion of lipid bilayers, consistent with the characteristics of smaller vesicles. These compositional differences indicate that ultracentrifugation enriches for smaller, membrane-dense vesicles with higher lipid content and lower protein load, whereas low-speed centrifugation retains larger, protein-rich structures and aggregates. Additionally, both protein and particle concentrations in the S100k supernatant were lower than in the S2k fraction following PDNV removal; nonetheless, a surprisingly high number of particles or aggregates remained detectable in the S100k sample.

### 3.2 Density-based separation of the PDNV isolates

Density gradient ultracentrifugation (DGUC) was conducted with two primary objectives: to separate vesicles from the P15k and P100k isolates based on their buoyant density and to purify them for downstream analyses. Each isolate was fractionated into 12 distinct layers (Figure 1C). To assess particle distribution and enhance separation from soluble components, two discontinuous iodixanol gradient configurations were tested (Figure 1B). Setup 1 used a linear gradient between 10% and 40%, while setup 2 employed a broader non-linear low-density slope (5%–40%).

**FIGURE 4:**
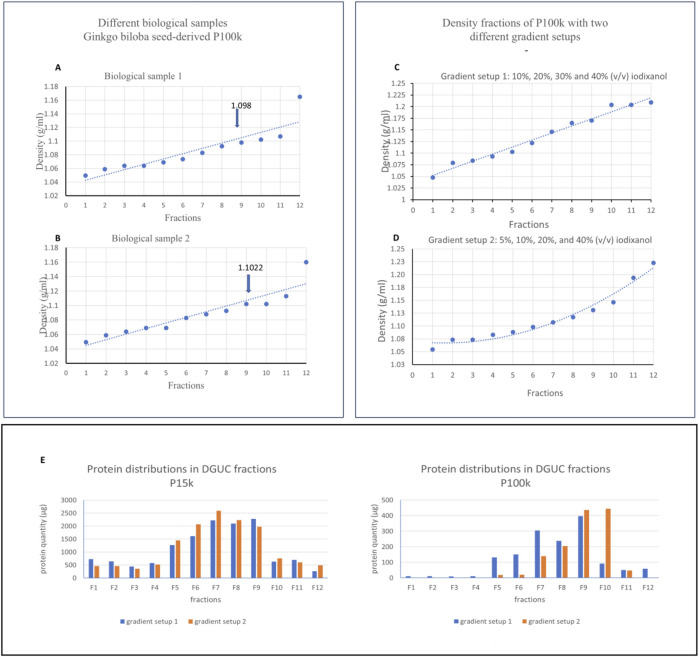
The densities measured in each fraction of two biological replicates **(A)** biological sample 1 and **(B)** biological sample 2, and experiments that applied two different gradient setups **(C, D)** for the separation of *Ginkgo biloba* seeds-derived P100k samples. Arrows show the density in visible fraction 9. A linear curve was fitted for gradient setup 1 **(A–C)**, whereas a polynomial curve was applied (dotted lines) for setup 2 **(D)**. Note: Very similar distribution curves were observed for the P15k density separations (data not shown). Chart **(E)** illustrates the protein distribution across the iodixanol density gradient fractions for both the P15K and P100K samples, using gradient setup 1 and setup 2.

**FIGURE 5:**
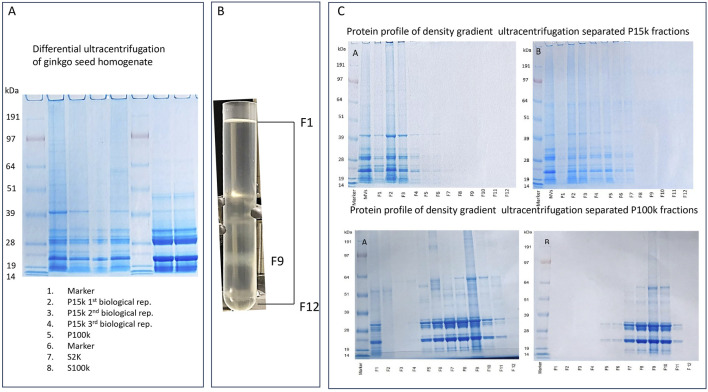
SDS-PAGE protein profiles of **(A)** *Ginkgo biloba* PDNV samples obtained in the differential ultracentrifugation (DUC) process: P15k (three biological replicates, lanes 2, 3, and 4), P100k (lane 5), and supernatants S2k (lane 7) and S100k (lane 8). **(B)** Representative image of the P100k sample with visible fractions at fraction 9 after the density gradient ultracentrifugation (DGUC) step. **(C)** SDS-PAGE protein profiles of the P15k and P100k fractions after the iodixanol DGUC using gradient setup 1 (left images) and setup 2 (right images). Fractions 1–12 were collected sequentially from the top to the bottom of the ultracentrifugation tube following DGUC. A 10 μL aliquot of each fraction was loaded onto the gel.

Figures 4A–D show the density measurements of iodixanol gradient fractions derived from the P15K and P100K samples. Following iodixanol DGUC, two distinct bands were consistently detected in both samples, indicating vesicle accumulation at specific density layers (see Figure 5B). The most prominent band was observed in fraction F9 (density: 1.098 g/mL), closely matching the typical density range of EVs isolated from mammalian cells. A second faint band appeared in fraction F2 (density: 1.079 g/mL). Two gradient setups were tested to improve separation and protein yield for both P15K and P100K samples (Figures 4C,D). While both setups yielded comparable results for P15K, the non-linear iodixanol gradient (5%, 20%, 30% and 40%) significantly increased protein recovery for P100K, concentrating most of the PDNVs in fractions F9 and F10 (Figure 4E).

### 3.3 SDS-PAGE protein profiles of ginkgo PDNVs

SDS-PAGE was performed to separate proteins and generate protein profiles for both the P15k and P100k PDNV samples (Figure 5). Figure 5A shows SDS-PAGE protein profiles of the two crude PDNV samples (P15k, lanes 2, 3, and 4 and P100k, lane 5) as well as the supernatants S2k (lane 7) and S100k (lane 8). There is a noteworthy similarity between the profiles of the two PDNV isolates. The S2k and S100k samples, on the other hand, show a less complex pattern than the PDNV samples and exhibit high-intensity low-molecular-mass proteins that, by mass, tentatively could be seed storage ginkgo proteins. Figure 5C displays the SDS-PAGE protein profiles of the F1–F12 fractions obtained by iodixanol DGUC from the P15k (upper images) and P100k (lower images) PDNVs. The gel images reveal a clear separation of vesicle fractions along the density gradient. Notably, fraction 9 (F9) of P15k exhibits a diffuse blue background, a characteristic feature of PDNVs, which may reflect increased lipid and/or RNA content in that specific vesicle population. This background is largely absent in other fractions, though faintly visible in F2. While several protein bands appear consistently across fractions, their relative intensities vary markedly, indicating differential protein abundance. Notably, the P15k PDNVs obtained from three separate seed batches, originating from different trees, exhibited consistent protein profiles, highlighting the reliability and reproducibility of the isolation procedure (Figure 4A).

In the P15k sample, the gel images demonstrate different vesicle populations across the gradient, with increasing density from F1 to F12. Notably, fraction 2 (F2) displays a diffuse blue background that is commonly associated with plant-derived nanovesicles (NVs) and potentially reflects increased lipid and/or RNA content. This feature is largely absent in other fractions, although it is still faintly visible in F3. While several protein bands appear across multiple fractions, their relative intensities vary substantially, indicating differential protein distribution among vesicle subpopulations.

It is noteworthy that the protein distribution patterns of DGUC-separated fractions of P100k and P15k PDNVs (Figure 5C) differ significantly. In the P100k samples, a distinct protein band was observed in fraction 9 at a density of 1.098. In contrast, the P15k samples showed prominent bands in fractions 2 and 3, with a density of 1.079. These observations suggest that high-density vesicles are predominantly enriched in the P100k fraction, whereas lower-density vesicle populations are present in the P15k fraction following DGUC.

### 3.4 Protein content of PDNVs isolated from the *Ginkgo biloba* seed homogenate

We employed LC-MS/MS-based proteomics to identify proteins present in the P100k PDNV sample purified by DGUC. Specifically, fractions 1, 3, 6, and 9 (Figure 5C), separated according to buoyant density, were selected for analysis. The list of proteins identified in these fractions is provided in Table 2. It is important to note that the GB proteome remains poorly characterized, which limits the number of proteins that can currently be identified with confidence. In fractions 1 and 3 (low-density fractions), only a single protein was detected: legumin (11S-globulin), a soluble seed storage protein belonging to the globulin family. These proteins are typically water- or salt-soluble and reside in the cytosol of plant seeds, rather than being membrane-bound. In fraction 6, legumin was again identified, along with ginnacin and the large subunit of ribulose bisphosphate carboxylase (RuBisCO). Given that the gradient was designed to isolate PDNVs, the presence of RuBisCO and ginnacin in this fraction may indicate co-purification of non-vesicular protein aggregates or dense organelle fragments, particularly from plastids or protein-rich compartments. The higher-density visible DGUC band in fraction 9 (Figure 5B) contained a broader protein profile, including legumin, ginnacin, and RuBisCO, as well as ATP synthase subunit alpha, ATP synthase protein MI25, and a putative heat shock protein 90, which is commonly detected in EV and PDNV proteomes and known to be expressed in ginkgo seeds. Additionally, the co-enrichment of catalase, phosphoenolpyruvate carboxylase (PEPC), and elongation factor 1-alpha (eEF1A) in this fraction likely reflects non-vesicular contamination, protein aggregation, or residual organellar debris. Notably, canonical plant EV marker proteins were not detected in our PDNV preparations.

**TABLE 2:** Identified proteins in fractions 1, 3, 6, and 9 from the S100K density gradient ultracentrifugation.

List of proteins identified in the *Ginkgo biloba* P100k PDNV sample			
Accession	Entry	Description	mW (Da)
P100k – F1			
Q39770	Q39770_GINBI	Legumin; 11S-globulin	51,418
P100k – F3			
Q39770	Q39770_GINBI	Legumin; 11S-globulin	51,418
P100k – F6			
Q39770	Q39770_GINBI	Legumin; 11S-globulin	51,418
Q39772	Q39772_GINBI	Ginnacin	51,444
A0A0B4UIU0	A0A0B4UIU0_GINBI	Ribulose bisphosphate carboxylase large chain (fragment)	48,647
P100k – F9			
Q39770	Q39770_GINBI	Legumin; 11S-globulin	51,418
Q39772	Q39772_GINBI	Ginnacin	51,444
I6N9F4	I6N9F4_GINBI	Ribulose bisphosphate carboxylase large chain	52,885
I2AP09	I2AP09_GINBI	Ribulose bisphosphate carboxylase large chain	52,713
A0A0D4BN81	A0A0D4BN81_GINBI	Ribulose bisphosphate carboxylase large chain (fragment)	29,340
Q39769	G3PC_GINBI	Glyceraldehyde-3-phosphate dehydrogenase_ cytosolic	36,745
A0A0N7AS68	A0A0N7AS68_GINBI	ATP synthase subunit alpha	54,697
Q9MM38	Q9MM38_GINBI	ATP synthase subunit alpha (fragment)	44,236
H1ZY27	H1ZY27_GINBI	Putative elongation factor 1-alpha (fragment)	17,759
Q8VXK8	Q8VXK8_GINBI	Phosphoenolpyruvate carboxylase (fragment)	41,339
H1ZY28	H1ZY28_GINBI	Putative heat shock protein 90 (fragment)	18,778
B8YNY2	B8YNY2_GINBI	Catalase	56,918
A0A0N7ASQ1	A0A0N7ASQ1_GINBI	ATP synthase protein MI25	22,219
Q39770	Q39770_GINBI	Legumin; 11S-globulin	51,418
Q39772	Q39772_GINBI	Ginnacin	51,444
I6N9F4	I6N9F4_GINBI	Ribulose bisphosphate carboxylase large chain	52,885

### 3.5 Exploring the protective effects of PDNVs isolated from *Ginkgo biloba* seeds in endothelial dysfunction by *in vitro* analysis

#### 3.5.1 Investigating the effect of *Ginkgo biloba* seed-derived PDNVs on human umbilical cord vein endothelial cell (HUVEC) viability

**FIGURE 6:**
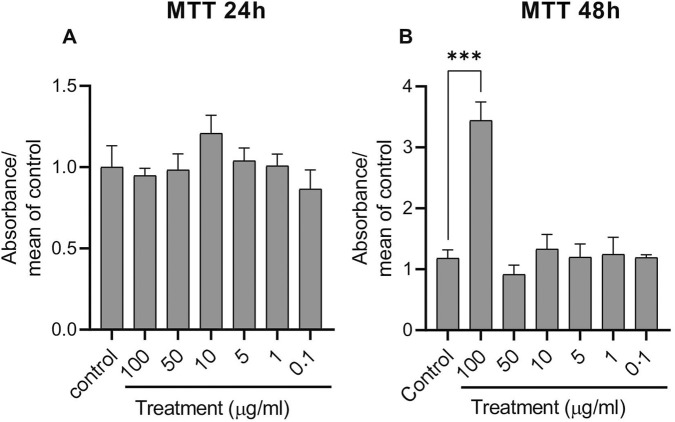
Effect of the applied ginkgo PDNVs on cell viability. Cells were seeded in 96-well plates (2 × 10^4^ cells/well) and treated at concentrations of 100 μg/mL, 50 μg/mL, 10 μg/mL, 5 μg/mL, 1 μg/mL, and 0.1 μg/mL for 24 h **(A)** or 48 h **(B)**. After treatment, the medium was removed, and cells were incubated with 100 µL of 0.5 mg/mL MTT solution at 37 °C for 3 h. Formazan crystals were dissolved in DMSO, and the absorbance of the solutions was measured at 565 nm using a CLARIOstar microplate reader (BMG Labtech, Ortenberg, Germany). Results are expressed as absorbance normalized to the mean of control values. Data represent the mean ± SD of three independent experiments performed in triplicate. Statistical analysis was performed using GraphPad Prism 8 software and a one-way ANOVA; ***p < 0.001 indicates significance. Abbreviations: PDNVs, plant-derived nanovesicles; DMSO, dimethyl sulfoxide.

First, we aimed to determine the optimal concentration of PDNVs that does not significantly affect the endothelial cell viability (Figure 6). HUVECs were exposed to PDNVs at different concentrations (0.1–100 μg/mL) for 24 h and 48 h. Cell viability was measured using the MTT assay. The MTT assay is a widely used colorimetric method to assess cell viability based on mitochondrial metabolic activity. Our results show that PDNV concentration ranging from 0.1 to 100 μg/mL did not significantly reduce HUVEC viability after 24 h (Figure 6A). A similar response was noticed when HUVECs were incubated for 48 h (Figure 6B). Interestingly, treatment with 100 μg/mL PDNVs significantly increases the mitochondrial activity (3.44 ± 0.33) compared to the control (1.18 ± 0.33) as measured by the MTT assay. However, increased mitochondrial activity detected by MTT does not necessarily indicate improved cell viability, as mitochondria play a central role in apoptosis. Hyperactivation of mitochondrial enzymes can occur during early apoptotic stages, which may explain the higher MTT signal at 100 μg/mL. To clarify whether this increase reflects enhanced cell health or apoptosis, we performed complementary fluorescent assays (Section 3.5.2).

#### 3.5.2 Investigation of the effects of *Ginkgo biloba* seed-derived PDNVs on apoptotic and necrotic processes in human umbilical vein endothelial cells (HUVECs)

**FIGURE 7:**
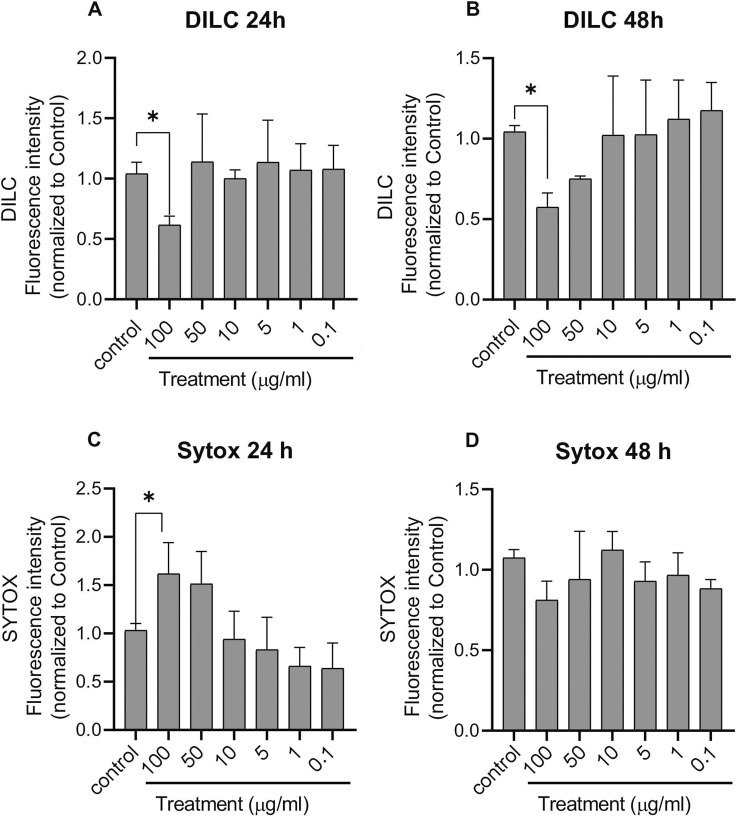
Effects of ginkgo PDNVs on apoptosis and necrosis. HUVEC cells were seeded in 96-well plates (2 × 10^4^ cells/well) and treated with PDNVs at 100 μg/mL, 50 μg/mL, 10 μg/mL, 5 μg/mL, 1 μg/mL, and 0.1 μg/mL for 24 h and 48 h. Apoptosis and necrosis were assessed using combined fluorescent DilC1(5) (apoptosis) and SYTOX Green (necrosis). **(A, B)** Apoptotic cell death after 24 h and 48 h, respectively. **(C, D)** Necrotic cell death at the same time points. Results are expressed as normalized fluorescence values relative to the control. Data represent the mean ± SD of three independent experiments. Statistical analysis was performed using one-way ANOVA in GraphPad Prism 8; *p ≤ 0.05 indicates significance.

We performed complementary fluorescent assays to assess whether concentrated PDNV application (100 μg/mL) exerts a cytotoxic effect detectable by the MTT assay. Specifically, we evaluated early apoptotic processes using DiLC1(5) staining and early necrotic processes using SYTOX Green labeling (Figure 7). HUVECs were exposed to PDNVs at concentrations ranging from 0.1–100 μg/mL for 24 h and 48 h.

One of the earliest indicators of apoptosis is the reduction of the mitochondrial membrane potential. Cyanine dyes such as DilC1(5) (1,1′,3,3,3′,3′-hexamethylindodicarbocyanine iodide) exhibit strong fluorescence when incorporated into membranes or bound to lipophilic biomolecules, although they are weakly fluorescent in an aqueous environment. This dye diffuses laterally within the cellular plasma membranes, providing uniform staining at optimal concentrations. DilC1(5) is widely used to assess mitochondrial membrane potential, where a loss of potential is reflected in a decrease in the infrared channel.

Our results show that PDNV treatment at 100 μg/mL induced apoptotic events within 24 h (Figure 7A). Similar effects were observed after 48 h, which aligns with the trend seen in the MTT assays (Figure 6B).

SYTOX Green fluorescent dye is commonly used to assess cell viability and necrosis. It cannot penetrate intact cell membranes but readily enters cells with compromised membranes, characteristic of necrotic or dead cells. The resulting fluorescent signal is exceptionally bright, making SYTOX Green particularly useful for detecting non-viable mammalian cells. In our study, we evaluated whether PDNV treatment induces necrosis in HUVECs (Figures 7C,D). After 24 h, treatment with 100 μg/mL PDNVs significantly increased SYTOX fluorescence (1.96 ± 0.61) compared to controls (1.02 ± 0.11), indicating necrotic processes (Figure 7C). Interestingly, no significant necrosis was observed after 48 h of treatment (Figure 7D).

#### 3.5.3 Investigation of the effect of ginkgo seed PDNVs on the intracellular ROS production of HUVEC

CM-H_2_DCFDA (chloromethyl-2′,7′-dichlorodihydrofluorescein diacetate) is a widely used indicator for detecting reactive oxygen species (ROS). After passive diffusion into cells, CM-H_2_DCFDA is deacylated by intracellular esterases, and its chloromethyl group reacts with intracellular thiols such as glutathione. Oxidation of the resulting compound generates a fluorescent product that becomes trapped within the cell, enabling long-term ROS monitoring. The detected fluorescence intensity is directly proportional to the intracellular ROS levels.

**FIGURE 8:**
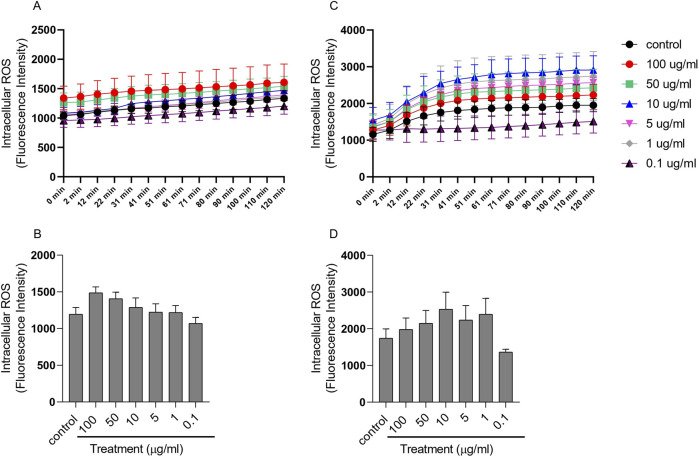
Effect of ginkgo PDNVs on ROS production. The cells were seeded in 96-well plates (2 × 10^4^ cells/well) and treated with PDNVs at 100 μg/mL, 50 μg/mL, 10 μg/mL, 5 μg/mL, 1 μg/mL, and 0.1 μg/mL concentrations for 1 h. ROS level was assessed using DCFDA (50 μM) staining for 30 min at 37 °C in the dark. After incubation, cells were washed twice with PBS to remove excess dye. Fluorescence was measured using a CLARIOstar microplate reader (BMG Labtech, Ortenberg, Germany) at 485 nm excitation and 530 nm emission. **(A, B)** Cell without H_2_O_2_ pre-treatment; **(C, D)** Cell pre-treated with H_2_O_2_ before PDNV exposure. Data represent mean ± SD. of three independent experiments. Statistical analysis was performed using the one-way ANOVA in GraphPad Prism, 8; *p ≤ 0.05; **p ≤ 0.01. ***p ≤ 0.001. Abbreviations: H_2_O_2_, hydrogen peroxide; PDNVs, plant-derived nanovesicles.

To determine the optimal PDNV concentration for reducing oxidative stress, we assessed ROS production in HUVECs under different treatment conditions (Figure 8). PDNV treatments alone did not significantly increase intracellular ROS levels compared to controls (Figures 8A–C). Under H_2_O_2_-induced oxidative stress (100 µmol), PDNVs exerted a protective effect, with 0.1 μg/mL showing the strongest reduction in ROS (Figure 8). Although 0.1 μg/mL PDNVs exhibited the strongest ROS reduction under oxidative stress, we selected 1 μg/mL for subsequent experiments because it provided consistent effects across multiple assays and ensured adequate PDNV internalization for mechanistic studies without cytotoxicity. Importantly, both concentrations were within the non-toxic range confirmed by MTT and fluorescent assays.

#### 3.5.4 Ginkgo seed PDNV treatment inhibited LPS-induced pro-inflammatory cytokine production on HUVECs

**FIGURE 9:**
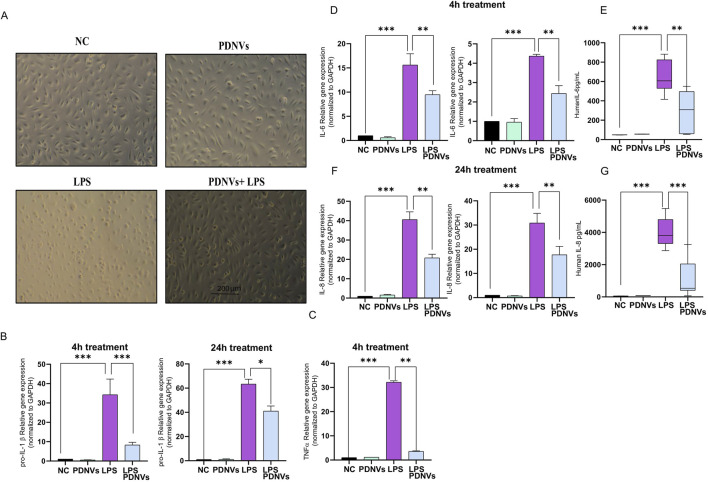
Effect of ginkgo PDNVs on inflammatory response in HUVECs. HUVEC-TERT cells were treated with PDNVs (1 μg/mL) alone and in combination with LPS (200 ng/mL) for 4 h and 24 h. **(A)** Morphological changes observed during treatments: negative control (NC) received culture medium only. **(B–F)** Gene expressions of pro-IL-1β, TNF-α, IL-6, and IL-8 were quantified by real-time PCR and normalized to GAPDH using the Livak method. **(E–G)** The IL-6 and IL-8 protein levels were measured by ELISA. Data represent the mean ± SD of three independent experiments. Statistical analysis was performed using one-way ANOVA in GraphPad Prism 8; *p ≤ 0.05; **p ≤ 0.01. ***p ≤ 0.001 indicate significance; PDNVs, plant-derived nanovesicles.

PDNVs have demonstrated notable anti-inflammatory properties in various cellular and animal models. In LPS-stimulated macrophages and microglia, *Ginkgo biloba* leaf extracts suppress the production of inflammatory mediators such as nitric oxide, prostaglandin E2, and pro-inflammatory cytokines. Therefore, we aimed to evaluate whether GB PDNVs can also exert positive effects on cell damage caused by LPS (Figure 9). To answer this question, endothelial cells were treated with PDNVs at a concentration of 1 μg/mL alone and in combination with LPS (200 ng/mL) for 4 h or 24 h. As shown in Figure 9A, LPS treatment induced major morphological changes in HUVECs. Notably, this phenomenon was not observed if LPS treatment was applied in combination with PDNVs.

In our experimental system, we first examined the mRNA expression of cytokines that are specifically and significantly increased during inflammation (Figures 9B,C,D–F). Relative mRNA transcriptional levels of IL-1β (Figure 9B), TNF-α (Figure 9C), IL-6 (Figure 9D), and IL-8 (Figure 9E) were investigated in each treatment condition. As expected, the relative mRNA expression of the investigated genes significantly increased as a result of LPS treatment compared to the control group (1 ± 0), IL-1β (35 ± 6), TNF-α (36 ± 7), IL-6 (18 ± 5), and IL-8 (41 ± 3). PDNV treatment at the 1 μg/mL concentration alone did not induce a significant increase in the tested pro-inflammatory cytokine gene expressions. Furthermore, PDNV treatment was able to alleviate the LPS-induced increases in all pro-inflammatory cytokine gene expressions. Similar inhibition effects were observed in the case of IL-6, IL-8, and IL-1β pro-inflammatory cytokine expression after both 4 h and 24 h of treatment. Interestingly, when we assessed TNF-α gene expression, we observed that after 24 h of treatment, LPS was not able to induce gene expression. TNF-α is an early-response cytokine, typically peaking within 1–6 h after LPS stimulation. By 24 h, its expression often returns to baseline due to negative feedback and post-transcriptional regulation.

To strengthen our findings, the protein levels of IL-8 and IL-6 were measured with ELISA from the cell culture supernatants after 24 h treatments (Figures 9E,F). PDNVs not only mitigated LPS-induced inflammation at the mRNA levels but also at the protein level. Specifically, PDNV treatment significantly reduced LPS-induced IL-6 (Figure 9E) as well as IL-8 (Figure 9F) protein expression compared to LPS alone.

#### 3.5.5 Effect of GB seed PDNVs on adhesion molecules and tight-junction protein expression of HUVECs after LPS treatment

Adhesion molecules and tight junction (TJ) proteins are critical regulators of endothelial barrier integrity and vascular homeostasis. During endothelial inflammation, molecules such as ICAM-1 and VCAM-1 become upregulated, promoting leukocyte adhesion and transmigration into tissues. Concurrently, disruption of TJ proteins like claudins and occludin compromises the endothelial barrier, leading to increased vascular permeability. These changes contribute to endothelial dysfunction, a hallmark of various inflammatory and cardiovascular diseases.

**FIGURE 10:**
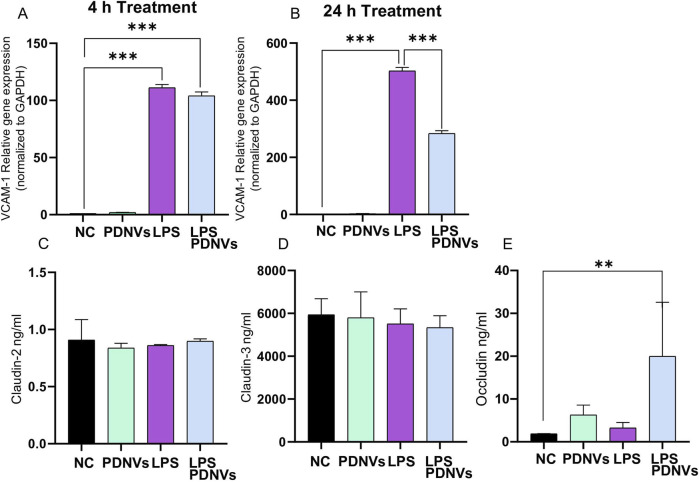
Effect of ginkgo PDNVs on LPS-induced regulation of adhesion molecules and tight junction proteins in HUVECs. HUVEC-TERT cells were treated with PDNVs (1 μg/mL) alone or in combination with LPS (200 ng/mL) for 4 h and 24 h. **(A, B)** Gene expression of VCAM-1 at 4 h and 24 h was quantified by real-time PCR and normalized to GAPDH using the Livak (2^−ΔΔCt^) method. **(C–E)** Protein levels of claudin-2, claudin-3, and occludin were measured using ELISA from cell culture supernatants. Data represent the mean ± SD of three independent experiments. Statistical analysis was performed using one-way ANOVA; *p ≤ 0.01; **p ≤ 0.001, ***p ≤ 0.0001 indicate significance. Abbreviations: PDNVs, plant-derived nanovesicles; LPS, lipopolysaccharide.

To investigate the effect of LPS exposure on adhesion molecules and tight junction proteins, HUVECs were incubated with LPS (100 ng/mL) in the absence or presence of PDNVs (Figure 10) for 4 h or 24 h. The mRNA levels of VCAM-1 were quantified by qRT-PCR (Figures 5A,B). After the 4-h treatment, LPS (110 ± 10) significantly increased VCAM-1 expression compared to the negative control (1 ± 0) (Figure 10A), and PDNV treatments did not mitigate this early induction. However, prolonged exposure to LPS (24 h) resulted in a more pronounced increase in VCAM-1 mRNA expression (Figure 10B), and under these conditions, PDNVs partially attenuated the LPS-induced upregulation.

The levels of TJ proteins after a 24 h treatment, including claudin-2, claudin-3, and occludin, were determined in HUVECs in the presence of PDNVs alone or in combination with LPS by ELISA (Figures 10C–E). Our data indicate that neither PDNV nor LPS treatments, alone or in combination, pronounced significant changes in claudin-2 (Figure 10C) or in claudin-3 (Figure 10D) expression levels. In contrast, occludin levels significantly increased following treatment with LPS and PDNVs (Figure 10E). Notably, LPS treatments did not reduce the protein levels of TJ under the experimental conditions.

#### 3.5.6 PDNVs mediated modulation of inflammatory and vasoregulatory pathways in endothelial cells following LPS stimulation

**FIGURE 11:**
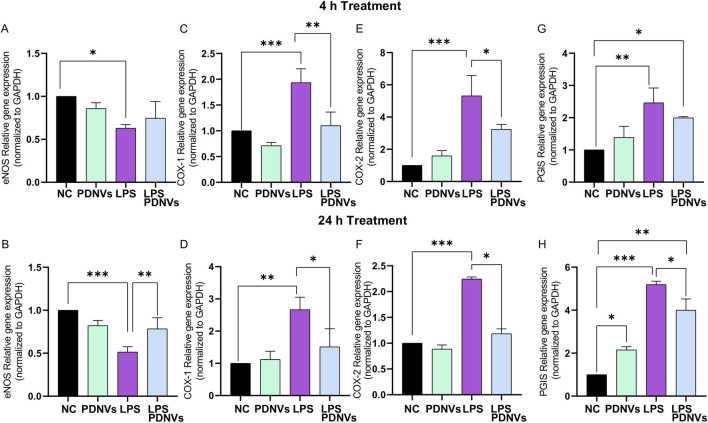
Effect of ginkgo PDNVs on endothelial homeostasis after LPS treatment. HUVEC-TERT cells were treated with PDNVs (1 μg/mL) alone or combined with LPS (200 ng/mL) for 4 h and 24 h. **(A, C, E, G)** Gene expression level of eNOS, COX-1, COX-2, and PGIS after 4 h; **(B, D, F, H)** the same targets after 24 h. Expression was quantified by real-time PCR, normalized to GAPDH using the Livak (2^−ΔΔCt^) method. Data represent the mean ± SD of three independent experiments. Statistical analysis was performed using one-way ANOVA; *p ≤ 0.05, **p ≤ 0.01, and ***p ≤ 0.001 indicate significance. Abbreviations: PDNVs, plant-derived nanovesicles; LPS, lipopolysaccharide.

The cytokines described above, due to their pleiotropic effect, play important roles in intracellular processes beyond inflammation, including endothelial vasorelaxation. Therefore, in this study, we investigated the effects of PDNVs on genes involved in human endothelial homeostasis (Figure 11).

HUVECs were treated with LPS (100 ng/mL) alone or in combination with PDNVs for 4 h or 24 h. First, we investigated the relative gene expression of endothelial nitric oxide synthase (eNOS), a key enzyme responsible for nitric oxide (NO) production and overall vascular homeostasis. During inflammation, NO synthesis is markedly impaired, contributing to the endothelial dysfunction. Our results showed that PDNVs can ameliorate LPS-induced damage by enhancing endothelium-dependent vasodilation through increased eNOS expression (Figure 11A).

LPS treatment significantly increases COX-1 mRNA compared to the controls at 4 h (Figure 11C). Notably, this increase was not observed when we used LPS combined with PDNVs. After 24 h, COX-1 expression remained markedly higher in LPS-treated cells compared to the control (Figure 11D), whereas PDNVs mitigated this LPS-induced elevation. Similar trends were observed for COX-2 expression at both 4 h and 24 h (Figures 11E,F). We also tested PGIS expression levels of PGIS after 4 h (Figure 11G) and 24 h (Figure 11H). LPS significantly increased PGIS expression at 4 h, and PDNVs did not reduce this early induction. However, after 24 h, PDNVs decreased the LPS-induced PGIS upregulation.

#### 3.5.7 Effects of PDNVs on the hemostatic and thrombotic mediators in endothelial cells

The endothelium produces a plethora of molecules, often with opposing functions that contribute to maintaining homeostasis, including vasodilatory, vasoconstrictive, procoagulant, anticoagulant, inflammatory, fibrinolytic, antifibrinolytic, oxidant, and antioxidant substances. Dysregulated endothelial function is associated with increased cardiovascular risk, often marked by altered levels of key coagulation and fibrinolytic factors, including thromboxane B_2_ (TXB_2_), von Willebrand factor (vWF), and plasminogen activator inhibitor-1 (PAI-1).

**FIGURE 12:**
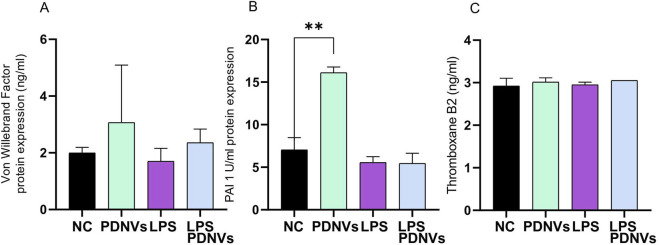
Effect of ginkgo PDNV treatment on endothelial function after LPS treatment. HUVEC-TERT cells were treated with PDNVs (1 μg/mL) alone or combined with LPS (200 ng/mL) for 24 h. **(A–C)** Von Willebrand factor, PAI-1, and thromboxane B_2_ were measured by ELISA. Data represent the mean ± SD of three independent experiments. Statistical analysis was performed using one-way ANOVA; *p ≤ 0.01 indicates significance. Abbreviations: PDNVs, plant-derived nanovesicles; LPS, lipopolysaccharide.

To evaluate the impact of PDNVs on these mediators, HUVECs were treated with PDNVs alone or in combination with LPS for 24 h, and protein levels were quantified by ELISA (Figure 12). Our results show no significant changes in vWF, PAI-1, or TXB_2_ levels following treatment with either PDNVs alone or in combination with LPS.

## 4 Discussion

Although GB seeds are widely used in traditional Chinese medicine, they remain significantly less studied than the leaf extract, particularly in the context of EV research. Seeds are also rarely employed as starting material for the isolation of PDNVs, despite their rich biochemical composition and therapeutic potential. In this study, we addressed this gap by developing an optimized protocol for isolating PDNVs from GB seeds, combining homogenization with DUC and iodixanol-based DGUC. This approach yielded a biomaterial enriched in nanovesicles, with both the P15K fraction (2.4 ± 0.3 mg protein per gram of seed) and the P100K fraction (0.28 ± 0.003 mg protein per gram of seed) successfully resolved using a tailored gradient setup (5%, 10%, 20%, and 40%). NTA and cryo-TEM confirmed the presence of nanometer-sized vesicular structures, validating the efficiency of our isolation strategy from this underexplored botanical source.

While GB is renowned for its antioxidant, anti-inflammatory, and vasoregulatory properties, most studies have focused on its leaf extracts, which are rich in flavonoids and terpenoids (Wang et al., 2021; Noor-E-Tabassum et al., 2022; Biernacka et al., 2023). In contrast, the seeds are less studied for these effects and are primarily noted for their phytochemical composition (Árva et al., 2024). Emerging evidence suggests that PDNVs may also contribute to these effects via bioactive protein cargo. However, proteomic studies of GB remain scarce and have primarily examined leaf tissue and pollen intine (Uvackova et al., 2014), with no comprehensive analysis of the ginkgo nut proteome to date. Notably, two low-molecular-weight antifungal proteins have been purified from GB seeds (Wang and Ng, 2000; Sawano et al., 2007), indicating latent bioactivity that warrants further exploration.

Historically, the vascular endothelium was viewed as a passive, semipermeable barrier facilitating molecular exchange between blood and interstitial tissues (Celermajer and Sydney, 1997). It is now recognized as a dynamic paracrine, endocrine, and autocrine organ essential for maintaining vascular tone, blood fluidity, and immune regulation (Grego et al., 2025). Endothelial dysfunction (ED), characterized by impaired vasodilation, inflammation, and pro-thrombotic signaling, is a central contributor to cardiovascular diseases (CVD), the leading cause of mortality worldwide. Despite the therapeutic relevance of GB, its seed-derived PDNVs have not been investigated in the context of ED, prompting the present study.

Our findings provide compelling evidence that PDNVs from GB seeds protect endothelial cells under inflammatory stress. Using HUVECs as a model, we demonstrated that GB PDNVs are well tolerated across a broad concentration range, with no significant cytotoxicity observed in MTT assays. Interestingly, high-dose GB PDNVs enhanced mitochondrial activity, prompting further investigation into apoptotic and necrotic pathways. Fluorescent assays revealed that while 100 μg/mL GB PDNVs induced early apoptotic and necrotic events, these effects were transient and dose-dependent, supporting the selection of 1 μg/mL as a safe and biologically active concentration for downstream experiments.

GB PDNVs did not increase intracellular ROS, even under oxidative stress conditions, indicating a favorable redox profile. More importantly, they significantly mitigated LPS-induced endothelial inflammation, as evidenced by reduced expression of key pro-inflammatory cytokines (IL-1β, TNF-α, IL-6, IL-8) at both mRNA and protein levels. This anti-inflammatory effect extended to adhesion molecules and TJPs: GB PDNVs attenuated VCAM-1 expression and increased occludin levels, indicating preserved barrier integrity. Furthermore, GB PDNVs modulated vasoregulatory pathways by restoring eNOS expression and suppressing COX-1, COX-2, and PGIS upregulation, thereby promoting endothelial homeostasis. Although no significant changes were observed in hemostatic mediators (TXB_2_, vWF, PAI-1), the overall profile supports a non-thrombogenic and vasoprotective role for GB PDNVs.

These functional outcomes are supported by proteomic analysis of GB PDNVs, which revealed a protein cargo enriched in legumin, ginnacin, RuBisCO, ATP synthase subunits, HSP90, catalase, PEPC, and eEF1A. Several of these proteins, particularly catalase, HSP90, and eEF1A, are known to modulate oxidative stress, inflammation, and cytoskeletal dynamics, offering mechanistic insights into the observed cellular effects. The presence of legumin and ginnacin may further contribute to antioxidant and immunomodulatory functions, while ATP synthase components suggest metabolic support. Although canonical plant EV markers were absent, the protein ensemble identified in GB PDNVs reflects a biologically active profile capable of influencing endothelial physiology.

These findings align with previous studies demonstrating that GB leaf extracts inhibit TNF-α-induced VCAM-1 expression and reduce ROS production in endothelial cells, partly through NF-κB and cAMP-mediated pathways (Chen et al., 2012; Piazza et al., 2019; Zhang and Yan, 2019). While those studies focused on leaf-derived compounds, our data suggest that GB seed-derived PDNVs may exert similar antioxidant and anti-inflammatory effects, potentially involving activation of the Nrf2 signaling cascade, a central regulator of vascular protection. Collectively, our results position GB seed-derived nanovesicles as promising candidates for vascular-targeted therapies, capable of mitigating endothelial inflammation and supporting vascular homeostasis through a multifaceted, protein-mediated mechanism.

We also acknowledge that PDNVs are intrinsically complex bionanomaterials, comprising heterogeneous vesicle populations (EVs and intracellular vesicles released during homogenization or cell rupture) together with co-purifying proteins, as confirmed by our proteomics analysis. These non-vesicular components are usually not fully separable from the vesicular fraction and may themselves exert bioactive effects, for example, by modulating inflammation or oxidative stress. Thus, the observed anti-inflammatory and vascular-protective responses may reflect the combined activity of vesicular and co-purifying protein components. Distinguishing their specific contributions remains challenging. Importantly, we also recognize that this compositional complexity is a defining feature of PDNVs as natural bionanomaterials and may contribute to their overall biological efficacy. Although this study provides comparative data on epithelial and endothelial barrier characteristics, including mRNA expression of adhesion molecules and protein expression of tight junction components, it has several limitations. We did not include functional migration or permeability assays, which would be necessary to directly assess barrier integrity and dynamic regulation in response to PDNV treatment. In addition, the biological effects of PDNVs at lower concentrations were not assessed. Although the selected concentration (1 μg/mL) allowed us to detect clear and reproducible responses, it remains unknown whether lower, potentially more physiologically relevant doses would elicit similar or distinct effects.

## Data Availability

The original contributions presented in the study are publicly available. This data can be found here: Submission details: ProteomeXchange title: Plant-Derived Nanovesicles (PDNVs) from Ginkgo biloba Seeds; ProteomeXchange accession: PXD072128. Project Webpage: https://www.ebi.ac.uk/pride/archive/projects/PXD072128.
